# Supplementary material for: Pulmonary maternal immune activation does not cross the placenta but leads to fetal metabolic adaptation
Source: Nat Commun. 2024 Jun 3;15:4711. doi: 10.1038/s41467-024-48492-x (PMC11148039; doi:10.1038/s41467-024-48492-x)
Supplement: Supplementary file 1 — Supplementary Information [file 41467_2024_48492_MOESM1_ESM.pdf]

SUPPLEMENTARY METHODS, NOTES AND FIGURES for

Pulmonary maternal immune activation does not cross the placenta but leads to fetal metabolic adaptation

Signe Schmidt Kjølner Hansen[1,2,3,*], Robert Krautz[1†], Daria Rago[1,2†], Jesper Havelund[4], Arnaud Stigliani[1,2], Nils J. Færgeman[4], Audrey Prézelin[5,6], Julie Rivière[7], Anne Couturier-Tarrade[5,6], Vyacheslav Akimov[4], Blagoy Blagoev[4], Betina Elfving[8], Ditte Neess[4], Ulla Vogel[3], Konstantin Khodosevich[2], Karin Sørig Hougaard[3,9,*,#], Albin Sandelin[1,2,*,#]

1 Department of Biology, University of Copenhagen, Denmark
2 Biotech Research and Innovation Centre (BRIC), University of Copenhagen, Denmark
3 National Research Centre for the Working Environment, Copenhagen, Denmark
4 Department of Biochemistry and Molecular Biology, University of Southern Denmark, Odense, Denmark
5 Université Paris-Saclay, UVSQ, INRAE, BREED, 78350 Jouy-en-Josas, France
6 Ecole Nationale Vétérinaire d'Alfort, BREED, 94700, Maisons-Alfort, France
7 Animal Genetics and Integrative Biology, INRAE, Université Paris Saclay, Jouy-en-Josas, France
8 Translational Neuropsychiatry Unit, Aarhus University, Aarhus, Denmark
9 Department of Public Health, University of Copenhagen, Denmark

[†]These authors contributed equally to this work
*Corresponding authors: Signe Schmidt Kjølner Hansen, signe.sk.hansen@sund.ku.dk; Karin Sørig Hougaard, ksh@nfa.dk; Albin Sandelin, albin@bio.ku.dk
[#] Shared last authorship

SUPPLEMENTARY METHODS 2
SUPPLEMENTARY NOTES 4
SUPPLEMENTARY FIGURES 6

SUPPLEMENTARY METHODS

Bronchoalveolar lavage

Lung inflammation in dams was assessed by collection of bronchoalveolar lavage fluid (BALf) followed by total and differential cell count[1]. Briefly, the trachea was cannulated by a 22 gauge needle equipped with a polyethylene catheter and the lungs were flushed twice by 0.8 ml of 0.9% NaCl through the trachea. Fluid was kept on ice until centrifugation at 400 × g at 4°C for 10 min. The pellet was resuspended in 100 µl medium (HAM F-12 with 1% penicillin/streptomycin and 10% fetal bovine serum). Aliquots of the cell suspension were used to determine numbers of live and dead cells by NucleoCounter (NC-200TM, Chemometec, Denmark), following manufacturer instructions. BALf cell composition (fractions of macrophages, lymphocytes, neutrophils and epithelial cells) was determined following centrifugation of 40 µl of suspension at 55xg for 4 min (Cytofuge 2, Statspin, TRIOLAB, Brøndby, Danmark) on to a microscope slide followed by fixation in 96% ethanol and staining with May-Grünwald-Giemsa. A minimum of 200 cells/slide were counted under a light microscope. All slides were randomized, blinded and scored by the same technician on the same day. By combining differential cell counts with cell numbers, the number of cells in each fraction could be calculated.

Saa3 RT-PCR analysis

We quantified lung mRNA expression levels of the acute phase response gene *Saa3*. RNA was isolated from 16–20 mg of tissue on Maxwell® 16 (Promega, USA) using Maxwell® 16 LEV simply RNA Tissue Kit (AS1280, Promega, USA) according to the manufacturer's protocol. RNA was eluted in 50 µl nuclease free (DEPC) water. cDNA was prepared from DNase treated RNA using Taq-Man® reverse transcription reagents (Applied Biosystems, USA) following the manufacturer's protocol. Total RNA and cDNA concentrations were measured on NanoDrop 2000c (ThermoFisher, USA). The Saa3 mRNA levels were determined using real-time RT-PCR with 18S RNA as reference gene. Each sample was run in triplicates on the ViiA7 Real-Time PCR (Applied Biosystems, USA). Saa3 primers and probe sequences were: forward: 5' GCC TGG GCT GCT AAA GTC AT 3′, reverse: 5' TGC TCC ATG TCC CGT GAA C 3′ and Saa3 probe: 5' FAM-TCT GAA CAG CCT CTC TGG CAT CGC T-TAMRA 3′. In all assays, TaqMan pre-developed mastermix (Applied Biosystems, USA) was used. Saa3 and 18S RNA levels were quantified in triplicates in separate wells. The relative expression levels of the target gene were calculated by the comparative method 2-ΔCt. Negative controls, where RNA had not been converted to cDNA (no template control), were included in each run. One sample, the plate control, was included in all Real-Time PCR analyses.

RNA-seq and lipidomics data integration

The integration of maternal and fetal liver RNA-Seq and lipidomics datasets was performed using the MixOmics package[2] through the implementation of two distinct models (one for the fetal liver and another for the maternal liver). Both models used the Diablo method[3] with the sparse partial least square (SPLS) approach to reduce the high dimensionality of our datasets. As we wanted to explore the connection between the mRNA and lipidomics mRNA data, and as the two omics were highly correlated in both datasets (> 0.95), we built a design matrix with 0.9 being the strength of relationship between the 2 omics. To obtain an optimal dimensionality explaining the majority of variance, initial models were constructed by integrating all available data for each dataset, employing the block.splsda function. The determination of ideal dimensionality was then achieved by executing the perf.diablo function on these initial models. This allowed us to reduce the fetal liver and the maternal liver to 1 and 3 components, respectively (these numbers were optimal to minimize the overall error rate with the maximum distance criteria).

In the SPLS framework, the *tune.block.splsda* function automatically identifies the necessary features (mRNA and lipids) within each component to explain most of the variation within a dataset. After running the *block.splsda* function again with these determined features, meaningful results were obtained for the fetal liver dataset (16 mRNAs and 8 lipids, as shown in Fig. S7A), but not for the maternal liver dataset as too few features were retained. Thus, for this setm we decided to artificially set the number of features to those in the fetal liver (16 mRNA and 8 lipids in one single component).

SUPPLEMENTARY NOTES

Note S1: Choice of LPS dose

LPS dose was chosen to model robust airway inflammation without causing excessive lung injury or preterm birth. In a pilot study, we administered 1.5, 4 or 7 ug LPS/animal or vehicle to non-pregnant mice. The lowest dose caused ~5% weight-loss after 24h (Fig. S1A) and was thus within the upper range of accepted body weight loss (10%) in short-term toxicity studies[4], indicative of maximum tolerable dose. At the two higher doses weight loss exceeded 10% after 24h. Pulmonary neutrophil-influx (a proxy for pulmonary inflammation) was similar across the three dosages after 24h, indicating saturation of neutrophil-influx already at 1.5 µg (Fig. S1B). As we were interested in placental transfer of inflammation and not outright toxicity, we chose the dose of 1 µg LPS/animal for our study.

Note S2: Blood cytokine correlation with cognate receptor expression in decidua and placenta

The Cxcl1 receptor *Cxcr2* was highly expressed in Ctrl decidua and placenta at all time points, while LPS increased Cxcl1 levels in maternal blood at 2h, suggesting a potential early interaction (Fig.2C, top middle). Notably, Cxcl1-Cxcr2 signaling in epithelial cells maintains immune-tolerance during pregnancy[5]. The Atypical chemokine receptor 2 (*Ackr2*) was consistently expressed at low levels in decidua and placenta (Fig.2C,top tight and bottom left). It scavenges inflammatory CC chemokines, including Ccl2, 7 and 11, which are chemoattractants for immune cells[6,7]. Since these chemokines were increased in maternal blood 2-12 h (Fig.2A) their ligand-receptor interactions may occur at 2-12h, which might prevent placental inflammation by restricting immune cell recruitment and infiltration.

Note S3: Development genes downregulated in fetal liver

At 5h, a number of genes associated with GO terms related to development were downregulated at 5h, including mesenchyme development, cell differentiation and ventricular and cardiac septum development (Fig.S5A). Of note, there is an overlap in genes responsible for liver and heart development. On gene level, we observed transient downregulation of growth factors and their positive regulators (e.g. *Smad4, Cited2, Ctnnb1, Fgfr2, Dand5*) and transcription factors (*Sox9, Lef1, Snai1/2*), mostly confined to 5h.

Note S4: Glycosylation genes downregulated in fetal liver

Genes associated with glycosylation-related GO-terms (Fig.S5B) were downregulated at 5h. Interestingly, some of these genes overlapped the downregulated glycosylation-associated genes in the placenta at 12h, including the *Dag1* associated genes *Pomgnt1-2*, *Pomt1-2* and *Fkrp*, and the Notch signaling-associated genes *Pofut1* and *Poglut1*. Additional dystroglycan and Notch-pathway associated genes were downregulated in fetal liver only, including *B3galnt2*, *Pomk* and *Poglut2*. Since dystroglycan glycosylation encourages a loosening in cell-cell adhesion and tissue permeability, we suggest that the dystroglycan decrease in fetal liver increases permeability and nutrient uptake during a state of scarcity. A group of genes related to glycosylation of glucose and carbohydrates (e.g., *B3gat3*, *Poglut2*, *B3galt6*, *Poglut3*, *St6gal1*) were also downregulated. Alterations

in glycosylation profiles of proteins involved in lipoprotein metabolism are associated with changes in their function[8] so we suggest that the alterations in carbohydrate and monosaccharide intermediates may also be an effect of the decreased cholesterol and fatty acid metabolism.

SUPPLEMENTARY FIGURES WITH LEGENDS

Figure S1

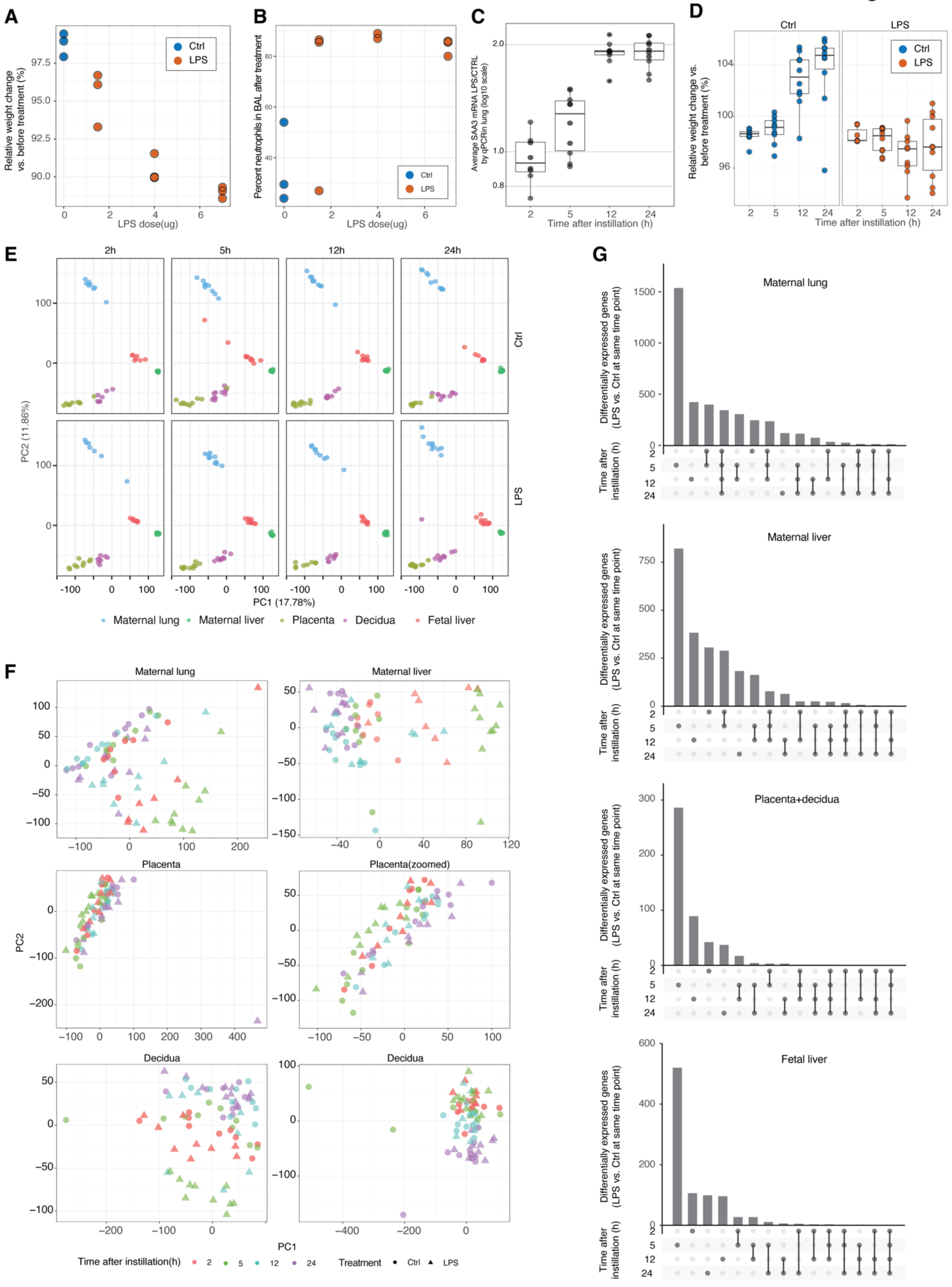

Figure S1

This figure expands Fig 1.

A: Weight loss vs. LPS dose. LPS instillation as in the main study was performed, but with varying dose. Y axis shows relative mice weight (%) vs. before treatment. X axis shows LPS dose. Dots indicate mice, colored by treatment. Mice were weighed before instillation and before euthanization (24h).

B: Neutrophils in Bronchoalveolar Lavage (BAL) vs. LPS dose. Neutrophil % in BAL were measured in the same mice as in A. Y axis shows % neutrophils in BAL. X axis shows as in A. Dots indicate mice, colored by treatment. Samples were collected 24h after exposure.

C: Saa3 expression change after LPS treatment. *Saa3* lung gene expression was measured after LPS/Ctrl instillation by real-time PCR. Dots on the Y axis show each LPS-instilled lung *Saa3* expression value divided by average Ctrl *Saa3* expression, on $\log_{10}$ scale. Box plots specifics as in Fig. 2E.

D: Mice relative weight change in the main experiment. Y axis shows % change vs. before treatment (Ctrl or LPS). Box plots and individual mice (dots) are shown, colored by treatment. X axis shows time after instillation. Box plots specifics as above.

E: Principal component analysis (PCA) of RNA-seq data, organized by instillation time and treatment. Axes show PC1-2 (% variance explained is shown). Columns and rows show time and treatment. Dots indicate dams or fetuses, colored by tissue.

F: PCA of RNA-seq data, organized by tissue. Panels shows RNA-seq data from specified tissues; for the placenta, a zoom-in view is also shown. Axes as in E. Dots indicate mice. Dot color shows time after instillation, shape shows treatment.

G: Differentially expressed (DE) genes and their overlap across time. Each upset plot shows the results for differential expression analysis in one tissue, as indicated on top. The top barplot in each plot shows the number of DE genes (LPS vs Ctrl, *FDR*<0.05) for one tissue on the Y axis. Time points are shown as rows below. Dots indicate the overlap of time point(s) plotted in the bar plot above. Lines between dots indicate genes DE across >1 time points.

Figure S2 page 1

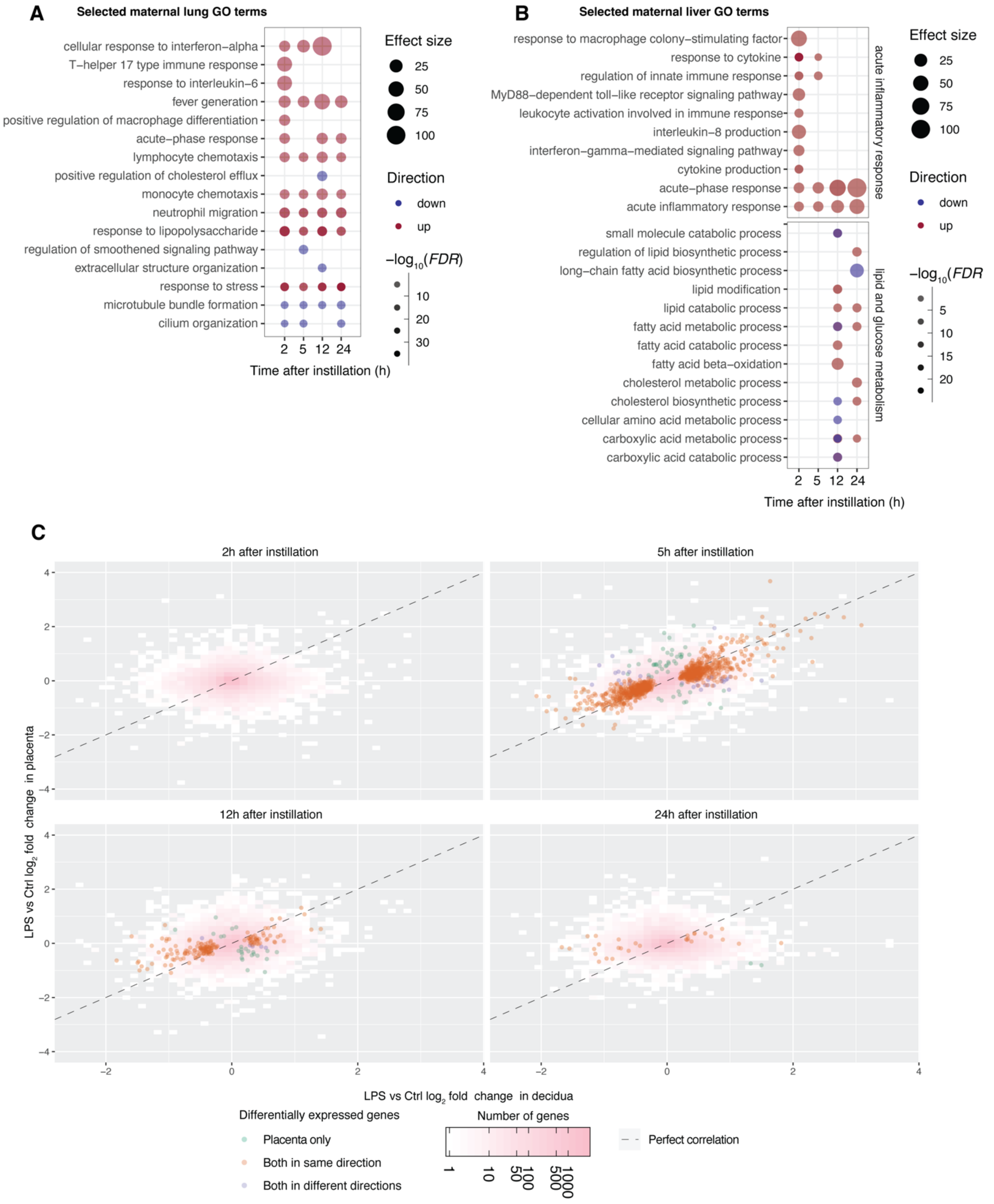

Figure S2 page 2

D

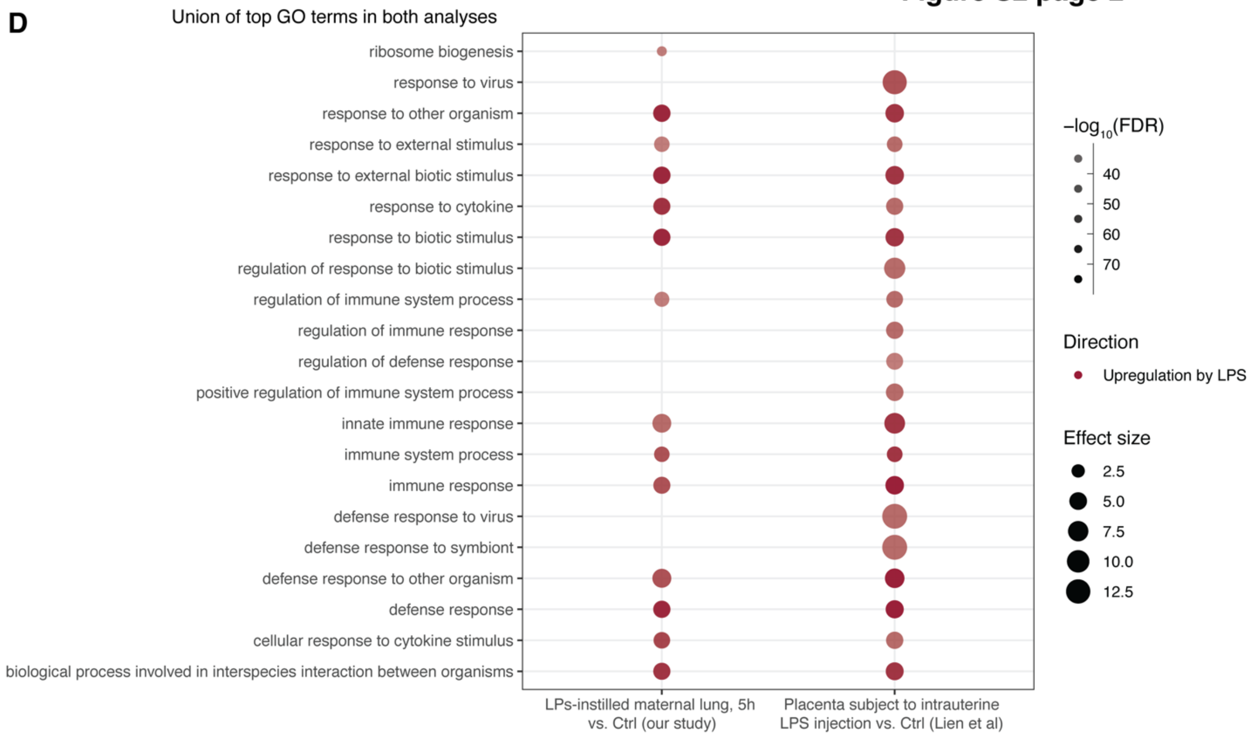

Figure S2
This figure expands Fig. 1
A: Gene ontology analysis of maternal lung RNA-seq data. X axis shows time after instillation. Rows show selected GO terms (see Table S1 for all terms). Dots show term over-representation in up- (red) or down-regulated (blue) genes following LPS instillation. Dot size and color intensity show enrichment effect size and statistical significance (-$\log_{10}$(*FDR*))
B: Gene ontology analysis of maternal liver RNA-seq data. Plot is organized as in H, but shows analysis of maternal liver RNA-seq data, and splits GO terms into two larger families, as indicated to the right.
C Correlation between LPS-instillation response between placenta and decidua. In each subplot, Y axis shows the LPS vs Ctrl $\log_2$FC expression change values of genes based on RNA-seq data from the placenta, while X axis shows corresponding $\log_2$FC values from decidua. Rectangle color shows the number of genes in each position of the plot. Dots show individual genes that are DE (LPS vs Ctrl, *FDR*<0.05) in placenta only, in both tissues in the same direction, and in both tissues but in opposite directions, as indicated by color. Each subplot shows one time point after instillation, as indicated on top. Dotted lines show perfect correlation (X=Y) lines.
D: Comparison of top LPS vs Ctrl GO terms in maternal lung following LPS instillation (our study) and intrauterine LPS injection (Lien et al). The plot is organized as in H, but shows two columns, one for each tissue comparison: selected GO terms are the union of the top-most significant GO terms in both analysis.

Figure S3 page 1

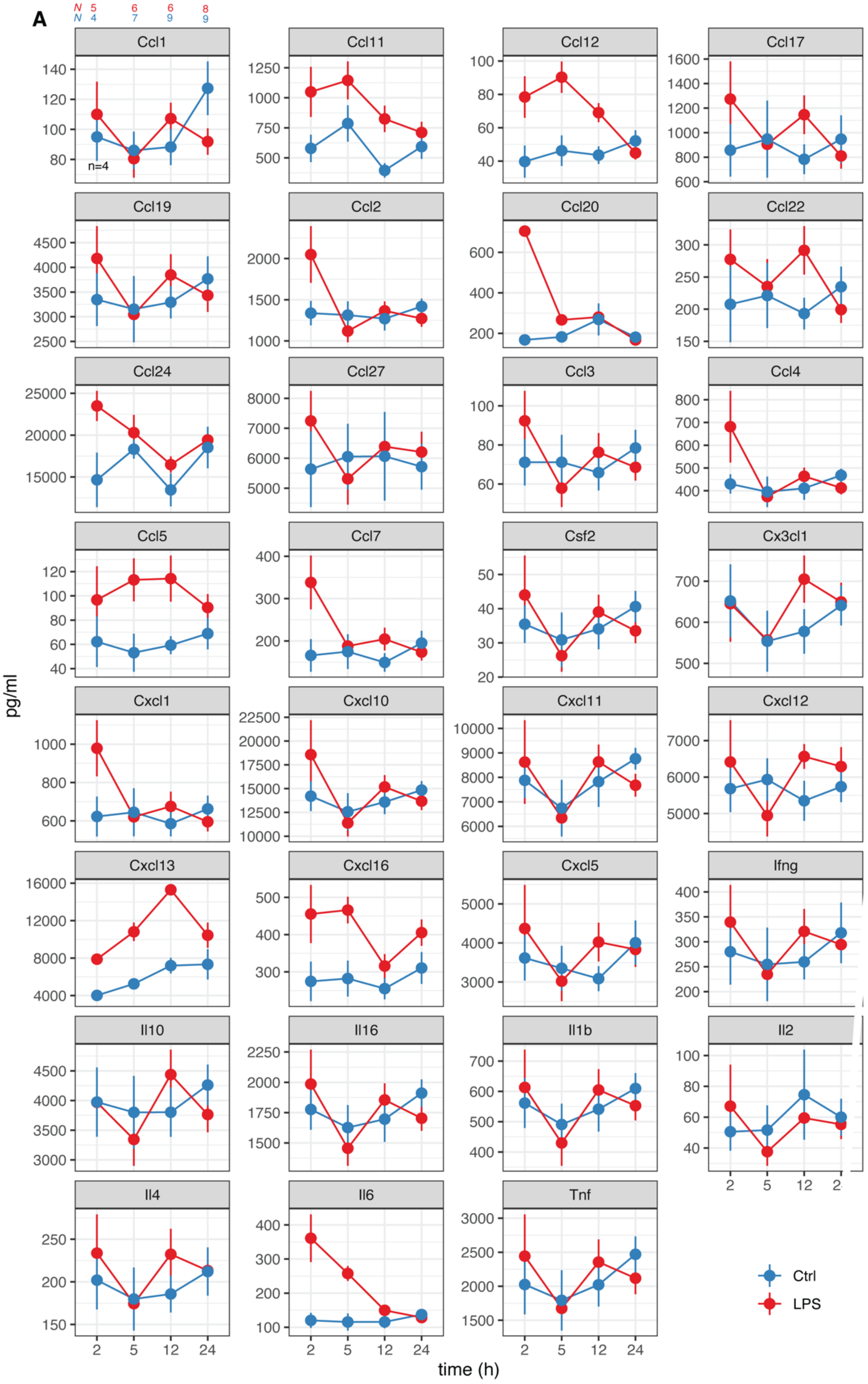

Figure S3 page 2

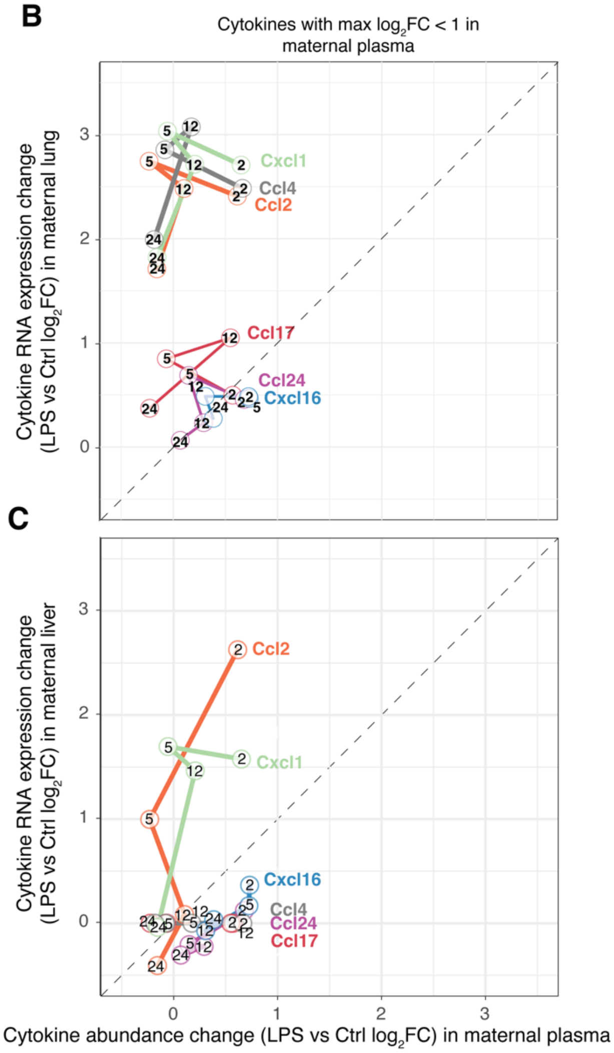

Fig.S3:

This figure expands Figure 2.

A: Absolute quantification of the 31 cyto- and chemokines measured in maternal plasma in Ctrl and LPS samples from all timepoints. The Y axis shows maternal plasma concentration (pg/mL). X axis show time after instillation. Dots show means of expression across replicates, error bars show SEM. Line and dot colors indicate treatment. Sample size shown for each group/timepoint in one plot. Number of samples are indicated on top ot the top left plot (same in all plots, colored by treatment as above)

B: Comparison of cytokine mRNA expression in maternal lung and protein levels in maternal plasma. The figure is organized as Fig 2A, but shows cytokines with small abundance changes in maternal plasma (max log_2FC <1).

C: Comparison of cytokine mRNA expression in maternal liver and protein levels in maternal plasma. The figure is organized as Fig 2B, but shows cytokines with small abundance changes in maternal plasma (max log2FC <1).

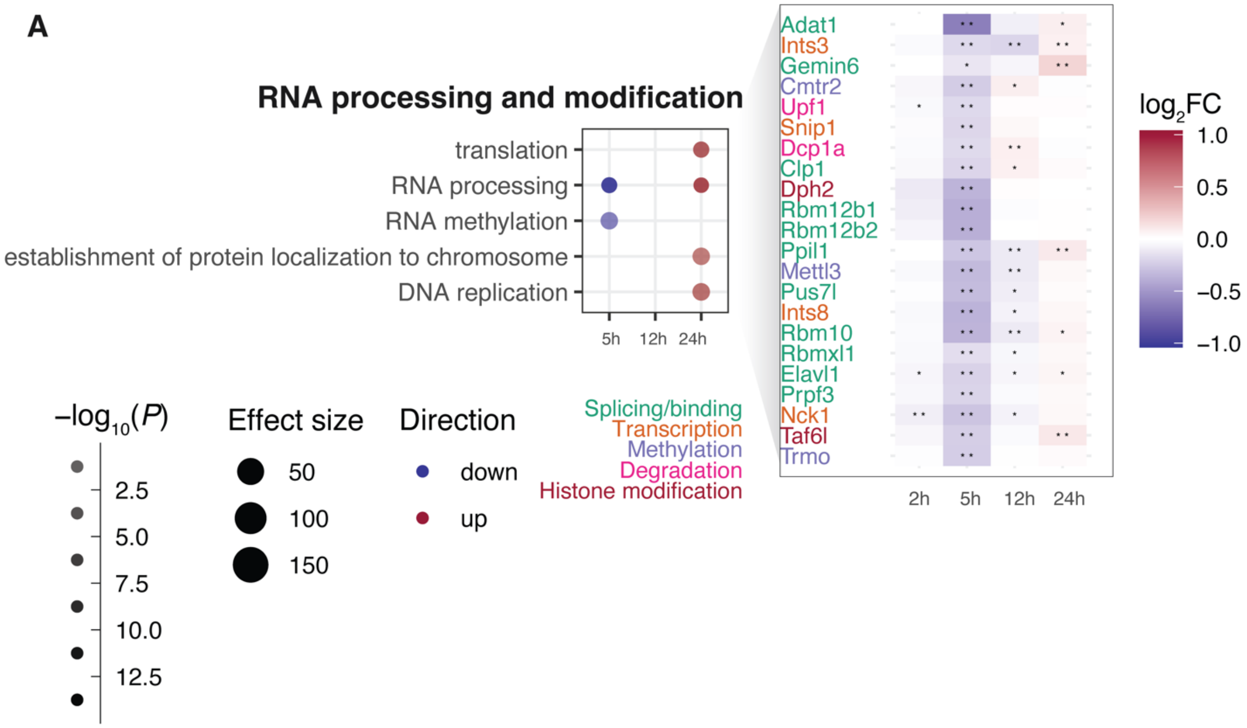

Fig.S4: Detailed analysis of placental gene expression change

This figure expands Fig.4 with an additional GO theme and associated genes, organized in the same way as the left and middle panels of Fig.4.

Figure S5

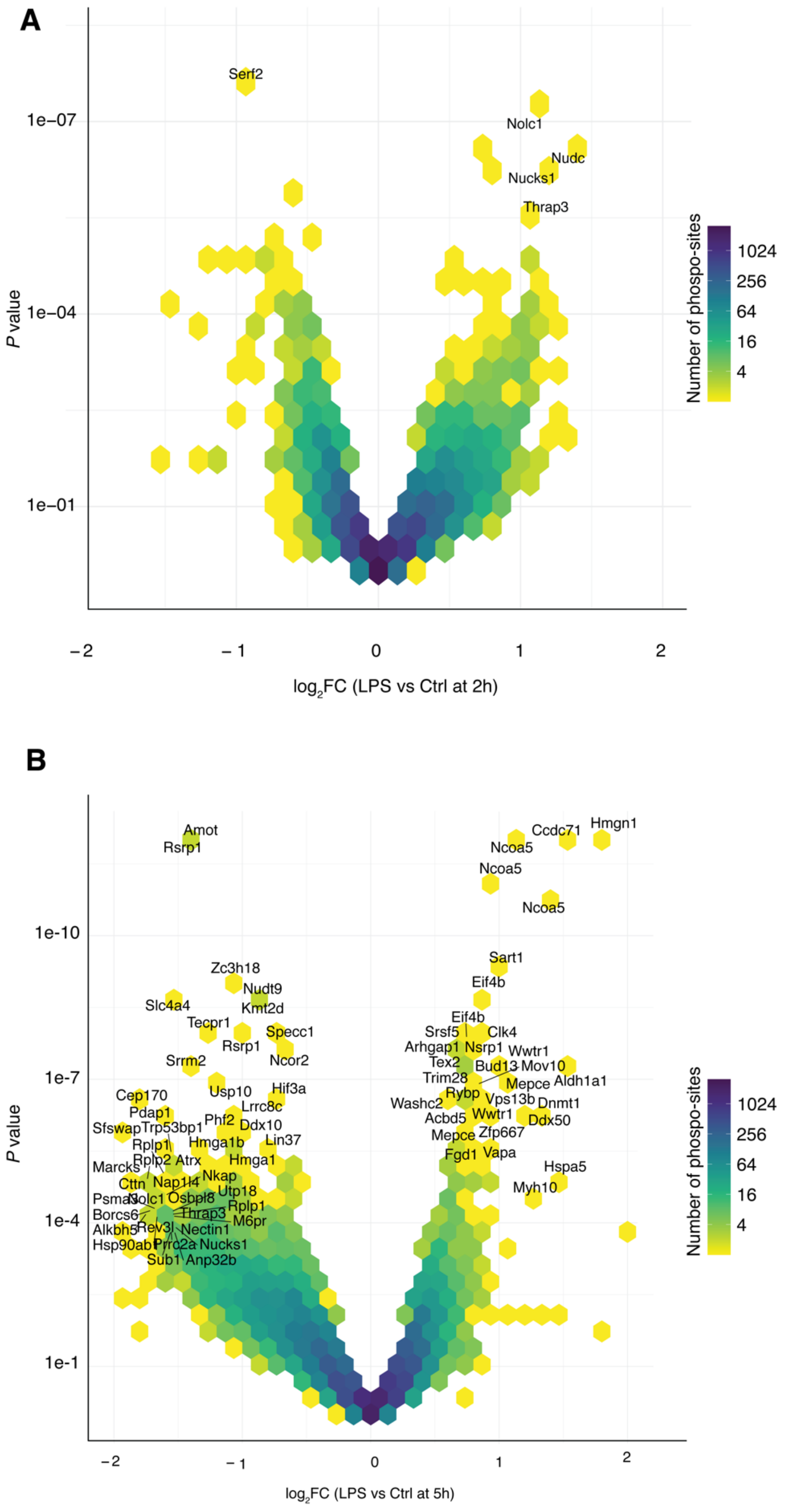
A
Serf2
Nolc1
Nudc
Nucks1
Thrap3
1e−07
1e−04
1e−01
P value
−2
−1
0
1
2
log2FC (LPS vs Ctrl at 2h)
Number of phospo-sites
1024
256
64
16
4
B
Amot
Rsrp1
Ccdc71
Hmgn1
Ncoa5
Ncoa5
Ncoa5
1e-10
Sart1
Eif4b
Zc3h18
Nudt9
Kmt2d
Slc4a4
Tecpr1
Specc1
Rsrp1
Ncor2
Srrm2
Eif4b
Srsf5
Clk4
Arhgap1
Nsrp1
Wwtr1
Tex2
Bud13
Mov10
Trim28
Mepce
Aldh1a1
1e-7
Usp10
Hif3a
Cep170
Lrrc8c
Pdap1
Phf2
Ddx10
Sfswap
Trp53bp1
Rplp1
Hmga1b
Lin37
Rplp2
Atrx
Hmga1
Marcks
Cttn
Nap1l4
Nkap
Utp18
Nolc1
Osbpl8
Rplp1
Borcs6
Thrap3
M6pr
Alkbh5
Rev3l
Nectin1
Hsp90ab1
Prrc2a
Nucks1
Sub1
Anp32b
Washc2
Rybp
Vps13b
Dnmt1
Acbd5
Wwtr1
Ddx50
Mepce
Zfp667
Fgd1
Vapa
Hspa5
Myh10
1e-4
1e-1
P value
−2
−1
0
1
2
log2FC (LPS vs Ctrl at 5h)
Number of phospo-sites
1024
256
64
16
4

Fig. S5: Phospoproteomics analysis of early placental time points

This figure expands Fig.6.

A: Phosphorylation site changes at 2h in placenta. The X axis shows LPS vs Ctrl log_2FC based on phosphoproteomics analysis (negative values correspond to loss of phosphorylation, positive to gain). The Y axis shows corresponding *P* values using $-log_{10}$ scale. Hexagon colors show the number of phospho-sites in a given region in the plot. Genes with substantial phosphosites are labeled, where genes mentioned in the main text are in bold.

B: Phosphorylation site changes at 5h in placenta. Plot is organized as in panel A, but shows data from 5h.

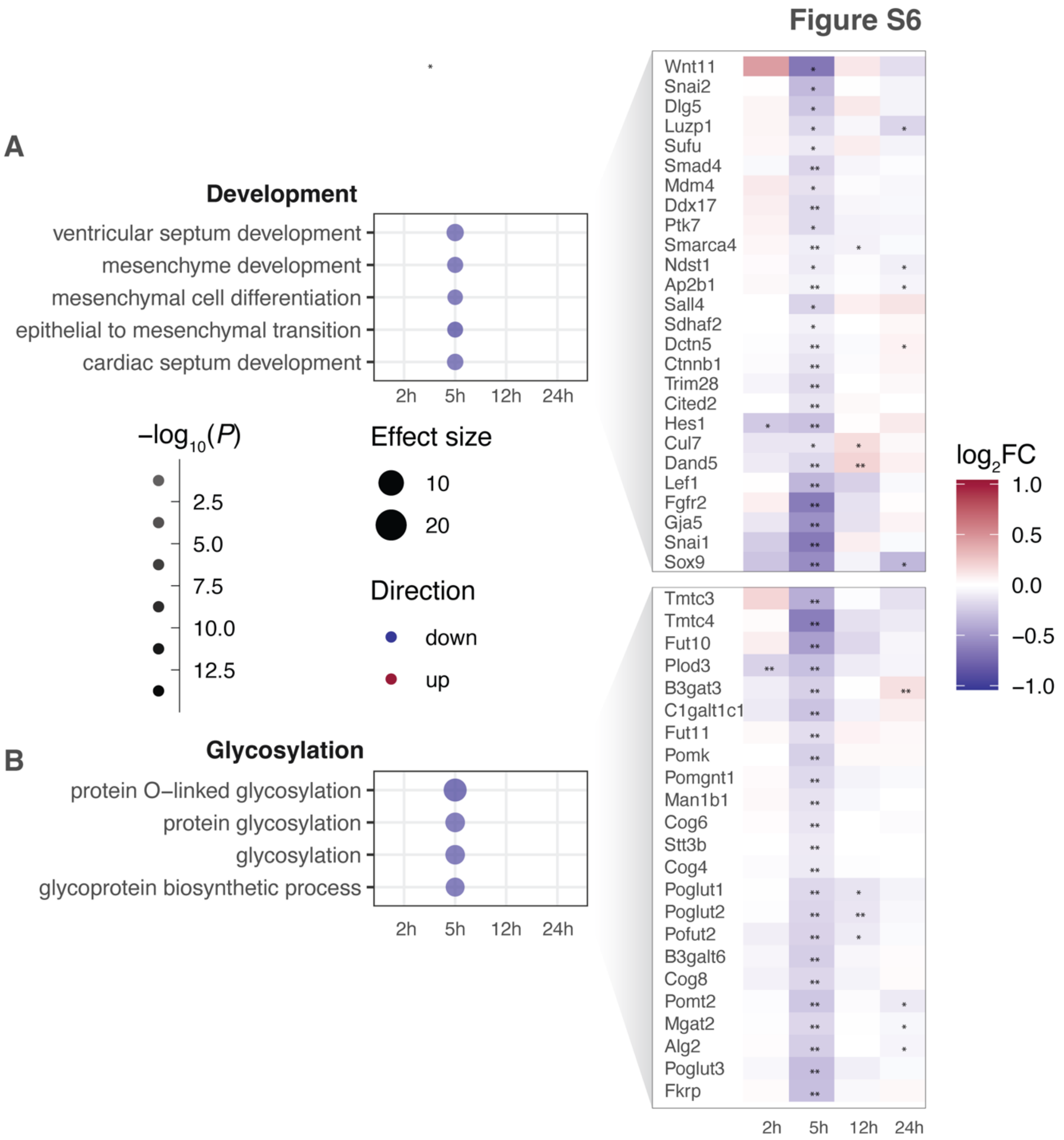

Fig.S6: Detailed analysis of fetal liver gene expression change
This figure expands Fig.7 with two additional GO themes and associated genes, organized in the same way as the left and middle panels of Fig.7.

Figure S7 page 1

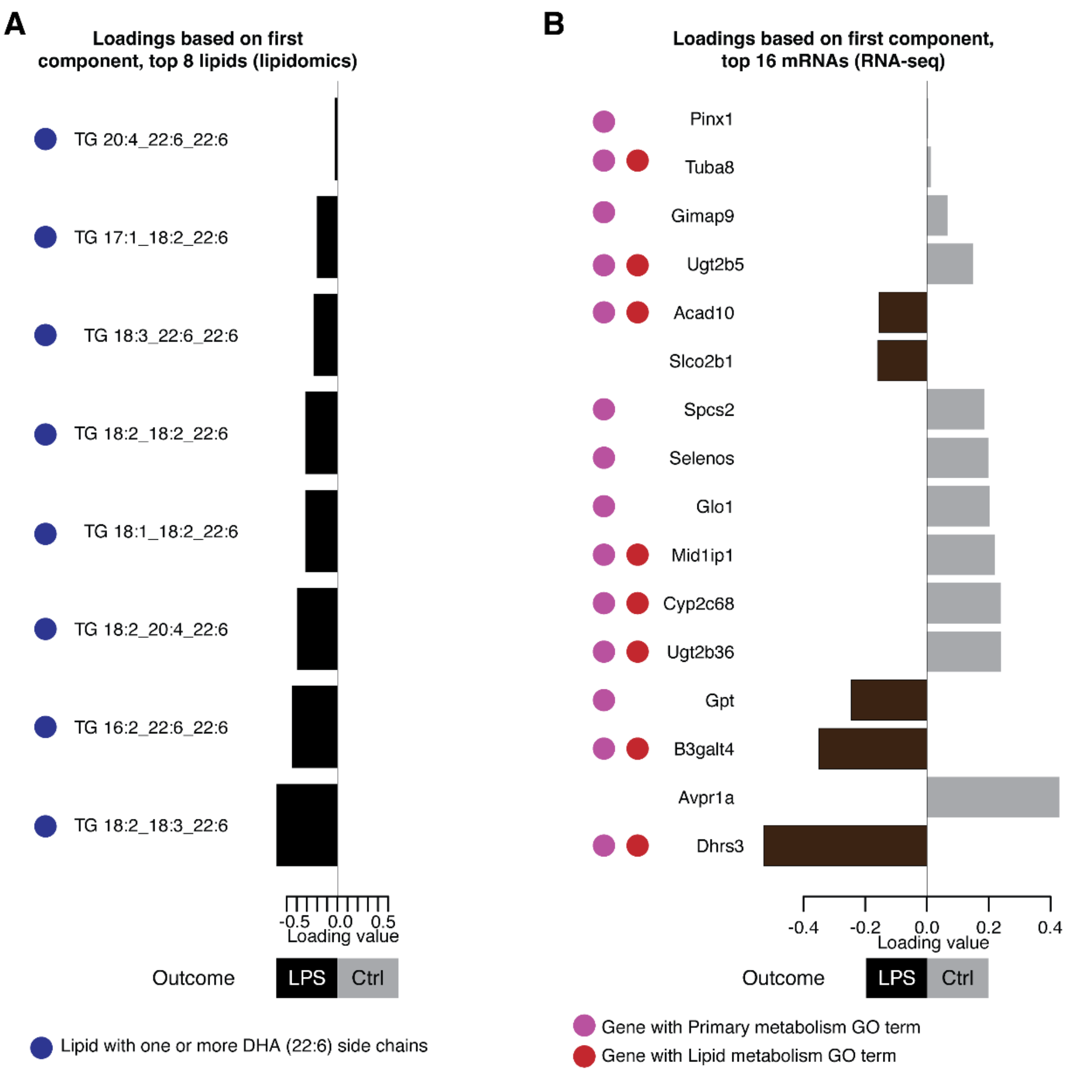

Fig S7, page 2

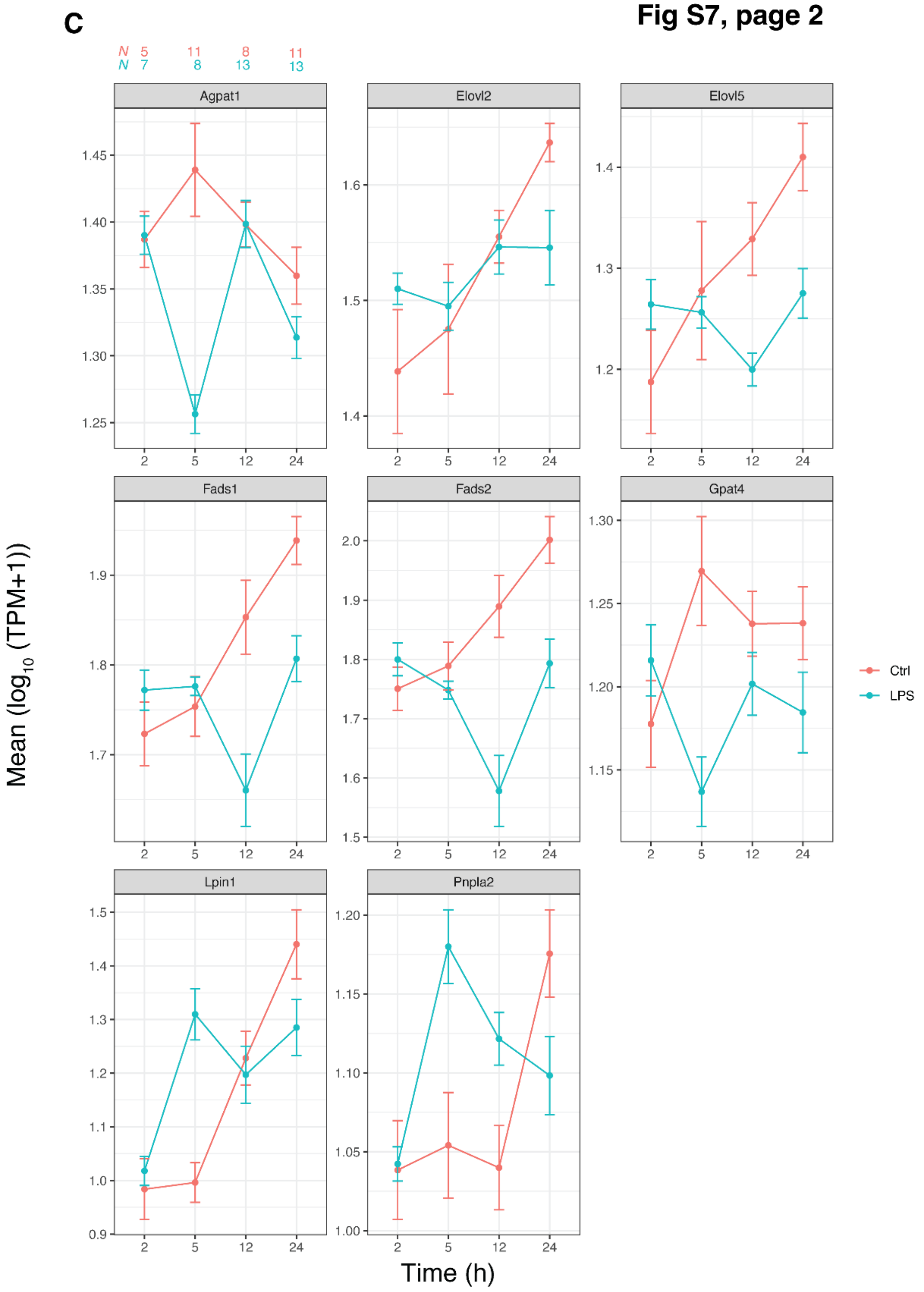

Fig. S7: Lipid- and associated RNA abundance related to synthesis of DHA-containing lipid species

This figure expands Fig.8.

A,B DIABLO discriminant analysis of lipidomics and RNA-seq data from fetal liver 12h after instillation. The plots collectively show a 'multi-omics signature' that best discriminates LPS vs Ctrl samples in fetal liver: A shows lipid species (lipidomics) while

B shows genes (RNA-seq). In each plot, X axis show loading values that indicate how informative a certain feature is for discrimination:a high positive values indicate that high abundance a gene or lipid is a signature of Ctrl samples while high negative loading values indicate that high abundance a gene or lipid is a signature of LPS samples. Circles to the left of lipids or genes show annotations as indicated by the legend.

C: Average mRNA expression of genes in Fig 6C. Images are organized as in the callout figures in Fig. 8C, but the Y axis shows $\log_{10}$(average RNA-seq TPM+1); gene names are shown on top. Number of samples are shown op top of the top left plot (same numbers in all plots, colors show treatment as above)

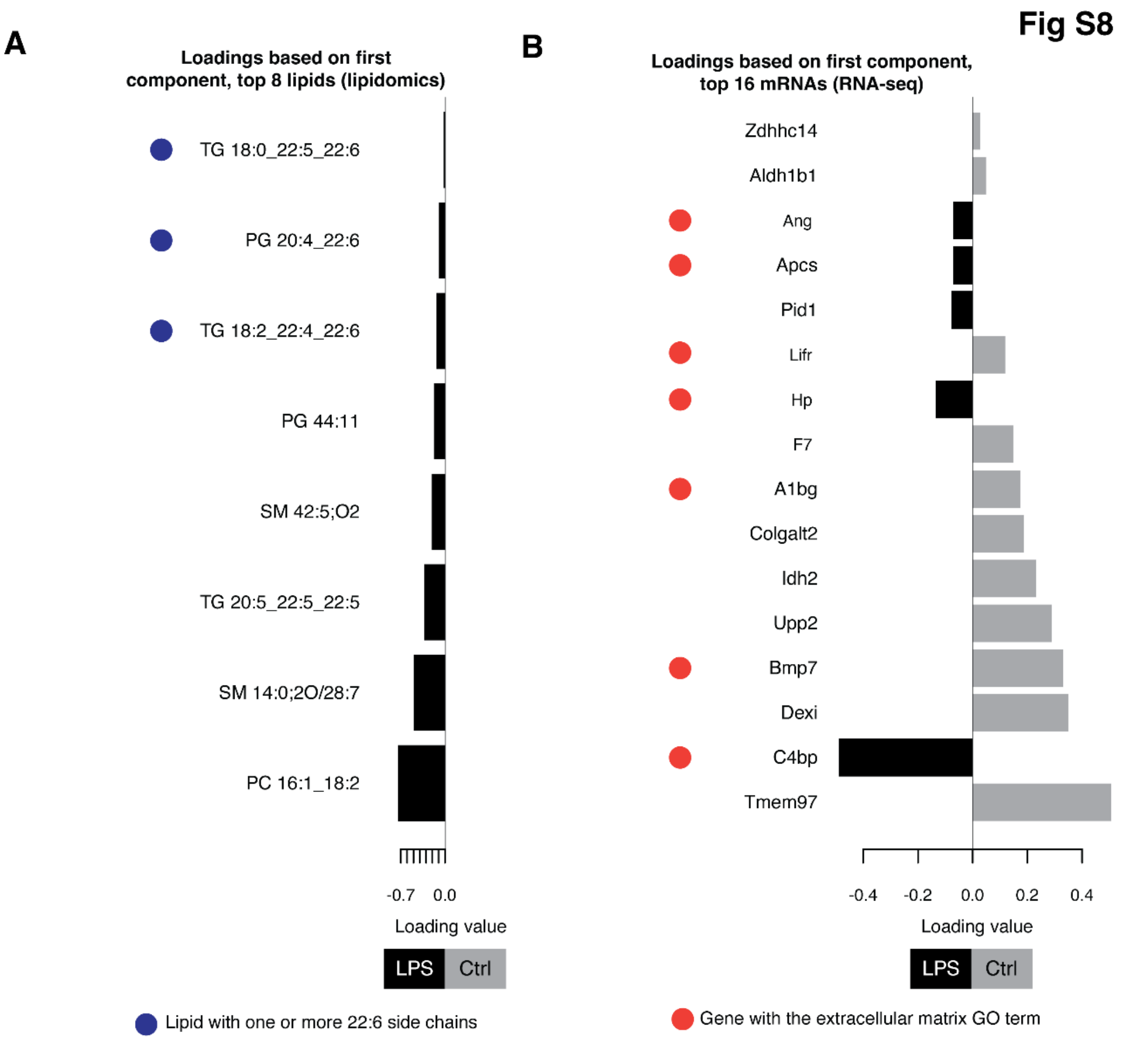

Fig. S8: Analysis of lipid- and RNA abundance in maternal liver and maternal plasma

This figure expands Fig.9.

A-B DIABLO discriminant analysis of lipidomics and RNA-seq data from maternal liver 12h after instillation. Figures are organized as in Fig.S7A,B but show analysis of maternal liver data.

Supplementary references

1. Umezawa, M. *et al.* Maternal inhalation of carbon black nanoparticles induces neurodevelopmental changes in mouse offspring. *Part. Fibre Toxicol.* **15**, 36 (2018).
2. Rohart, F., Gautier, B., Singh, A. & Lê Cao, K.-A. mixOmics: An R package for ’omics feature selection and multiple data integration. *PLoS Comput. Biol.* **13**, e1005752 (2017).
3. Singh, A. *et al.* DIABLO: an integrative approach for identifying key molecular drivers from multi-omics assays. *Bioinformatics* **35**, 3055–3062 (2019).
4. Chapman, K. *et al.* A global pharmaceutical company initiative: an evidence-based approach to define the upper limit of body weight loss in short term toxicity studies. *Regul. Toxicol. Pharmacol.* **67**, 27–38 (2013).
5. Kang, X. *et al.* CXCR2-Mediated Granulocytic Myeloid-Derived Suppressor Cells’ Functional Characterization and Their Role in Maternal Fetal Interface. *DNA Cell Biol.* **35**, 358–365 (2016).
6. Bonecchi, R. & Graham, G. J. Atypical Chemokine Receptors and Their Roles in the Resolution of the Inflammatory Response. *Front. Immunol.* **7**, 224 (2016).
7. Massara, M. *et al.* ACKR2 in hematopoietic precursors as a checkpoint of neutrophil release and anti-metastatic activity. *Nat. Commun.* **9**, 676 (2018).
8. Pirillo, A., Svecla, M., Catapano, A. L., Holleboom, A. G. & Norata, G. D. Impact of protein glycosylation on lipoprotein metabolism and atherosclerosis. *Cardiovasc. Res.* **117**, 1033–1045 (2021).
