# Supplementary material for: Pulmonary maternal immune activation does not cross the placenta but leads to fetal metabolic adaptation
Source: Nat Commun. 2024 Jun 3;15:4711. doi: 10.1038/s41467-024-48492-x (PMC11148039; doi:10.1038/s41467-024-48492-x)
Supplement: Supplementary file 3 — Reporting Summary [file 41467_2024_48492_MOESM3_ESM.pdf]

nature portfolio

Corresponding author(s): Schmidt Kjølner Hansen, Sørig Hougaard, Sandelin

Last updated by author(s): 27 feb 2024

Reporting Summary

Nature Portfolio wishes to improve the reproducibility of the work that we publish. This form provides structure for consistency and transparency in reporting. For further information on Nature Portfolio policies, see our Editorial Policies and the Editorial Policy Checklist.

Statistics

For all statistical analyses, confirm that the following items are present in the figure legend, table legend, main text, or Methods section.

n/a	Confirmed	
☐	☐	The exact sample size (n) for each experimental group/condition, given as a discrete number and unit of measurement
☐	☒	A statement on whether measurements were taken from distinct samples or whether the same sample was measured repeatedly
☐	☒	The statistical test(s) used AND whether they are one- or two-sided<br>*Only common tests should be described solely by name; describe more complex techniques in the Methods section.*
☐	☒	A description of all covariates tested
☐	☒	A description of any assumptions or corrections, such as tests of normality and adjustment for multiple comparisons
☐	☒	A full description of the statistical parameters including central tendency (e.g. means) or other basic estimates (e.g. regression coefficient) AND variation (e.g. standard deviation) or associated estimates of uncertainty (e.g. confidence intervals)
☐	☒	For null hypothesis testing, the test statistic (e.g. F, t, r) with confidence intervals, effect sizes, degrees of freedom and P value noted<br>*Give P values as exact values whenever suitable.*
☒	☐	For Bayesian analysis, information on the choice of priors and Markov chain Monte Carlo settings
☒	☐	For hierarchical and complex designs, identification of the appropriate level for tests and full reporting of outcomes
☐	☒	Estimates of effect sizes (e.g. Cohen's d, Pearson's r), indicating how they were calculated

Our web collection on statistics for biologists contains articles on many of the points above.

Software and code

Policy information about availability of computer code

Data collection	None
Data analysis	Multiqc v 1.1 and Segtk v 1.2 for for RNA-seq QC. Salmon v2 for mapping. R 4.0 was used for all statistical analyses . R Code and parameters are available at https://github.com/signehansen/inflammation_to_metabolism. QuPath 0.5.0 was used for imaging analysis. FAIMS-MzXML-Generator v1 was used for TMTPro correction for mass-spec data. MetabolAnalyze (version 1.3.1) was used to anlayze lipidomics data.

For manuscripts utilizing custom algorithms or software that are central to the research but not yet described in published literature, software must be made available to editors and reviewers. We strongly encourage code deposition in a community repository (e.g. GitHub). See the Nature Portfolio guidelines for submitting code & software for further information.

Data

Policy information about availability of data

All manuscripts must include a data availability statement. This statement should provide the following information, where applicable:

- Accession codes, unique identifiers, or web links for publicly available datasets
- A description of any restrictions on data availability
- For clinical datasets or third party data, please ensure that the statement adheres to our policy

The RNA-seq data generated in this study have been deposited in the GEO database under accession code GSE224116, https://www.ncbi.nlm.nih.gov/geo/query/

acc.cgi?acc=GSE224116. The proteomics raw data generated in this study have been deposited in the ProteomeXchange Consortium via the PRIDE partner repository under accession code PXD039402. The lipidomics raw data generated in this study has been deposited in the Metabolomics Workbench database under accession code PR001943 http://dx.doi.org/10.21228/M8K43P. Source data used for images are provided with this paper. The Lien et al RNA-seq data36 used in this study are available in the GEO database under accession code GSE151728, https://www.ncbi.nlm.nih.gov/geo/query/acc.cgi?acc=GSE151728.

Research involving human participants, their data, or biological material

Policy information about studies with human participants or human data. See also policy information about sex, gender (identity/presentation), and sexual orientation and race, ethnicity and racism.

Reporting on sex and gender	No human data.
Reporting on race, ethnicity, or other socially relevant groupings	NA
Population characteristics	NA
Recruitment	NA
Ethics oversight	NA

Note that full information on the approval of the study protocol must also be provided in the manuscript.

Field-specific reporting

Please select the one below that is the best fit for your research. If you are not sure, read the appropriate sections before making your selection.

☒ Life sciences ☐ Behavioural & social sciences ☐ Ecological, evolutionary & environmental sciences

For a reference copy of the document with all sections, see nature.com/documents/nr-reporting-summary-flat.pdf

Life sciences study design

All studies must disclose on these points even when the disclosure is negative.

Sample size	Initial sample size per group and time point 10 mice. No power analysis due to no knowledge of variance in advance, but based on previous experience and given that the mice are genetically homogenous, plus expected dropouts (see exclusions)
Data exclusions	Only pregnant mice included, resulting in 7-9 mice per group and time point.
Replication	Reprodicibility measurements based on RNA-seq in figure S1 (PCA)
Randomization	Random assignment of mice to treatment groups and sampling time point.
Blinding	Yes

Reporting for specific materials, systems and methods

We require information from authors about some types of materials, experimental systems and methods used in many studies. Here, indicate whether each material, system or method listed is relevant to your study. If you are not sure if a list item applies to your research, read the appropriate section before selecting a response.

Materials & experimental systems

n/a	Involved in the study	
☐	☒	Antibodies
☒	☐	Eukaryotic cell lines
☒	☐	Palaeontology and archaeology
☐	☒	Animals and other organisms
☒	☐	Clinical data
☒	☐	Dual use research of concern
☒	☐	Plants

Methods

n/a	Involved in the study	
☒	☐	ChIP-seq
☒	☐	Flow cytometry
☒	☐	MRI-based neuroimaging

Antibodies

Antibodies used	ATF-4 (1:100, Abcam/ab31390, Anti-rabbit) , SOCS3 (1:800, Invitrogen/PA5-87485) . For SOCS3, sections were coated with a biotinylated secondary antibody (dilution 1:500, Anti-rabbit Poly-HRP-IgG)
Validation	Commerical antibodoes are tested by the suppliers, see:<br>https://www.abcam.com/en-dk/products/primary-antibodies/atf-4-antibody-ab31390<br>https://www.thermofisher.com/antibody/product/SOCS3-Antibody-Polyclonal/PA5-87485

Animals and other research organisms

Policy information about studies involving animals; ARRIVE guidelines recommended for reporting animal research, and Sex and Gender in Research

Laboratory animals	Nulliparous C57BL/6JRj mice from Janvier, Saint Berthevin Cedex, France
Wild animals	NA
Reporting on sex	Female only, maternal and fetal. Fetus sex was determined by qPCR of genomic DNA (see Methods)
Field-collected samples	NA
Ethics oversight	All animal procedures followed the guidelines for care and handling of laboratory animals established by the EC Directive 86/609/EEC and Danish regulation (Danish Ministry of Justice, Experimental Animal Inspectorate, permit 2015–15–0201-00569). The local animal welfare committee approved the specific protocol prior to the study.

Note that full information on the approval of the study protocol must also be provided in the manuscript.

Plants

Seed stocks	NA
Novel plant genotypes	NA
Authentication	NA
